# Gyrate atrophy of the choroid and retina with hyper-ornithinemia responsive to vitamin B6: a case report

**DOI:** 10.1186/1752-1947-1-27

**Published:** 2007-06-12

**Authors:** Alireza Javadzadeh, Davood Gharabaghi

**Affiliations:** 1 Retina Service, Department of Ophthalmology, Nikookari Hospital – Drug of Applied research center, Tabriz University of Medical Sciences, Tabriz, Iran; 2 Strabismus Service, Department of Ophthalmology, Nikookari Hospital, Tabriz University of Medical Sciences, Tabriz, Iran

## Abstract

**Background:**

Gyrate atrophy of the retina and choroid is a rare autosomal recessive inherited disease, characterized by progressive chorioretinal atrophy that results in progressive deterioration of peripheral and night vision and leading to blindness.

**Case presentation:**

This report presents a case of a 28-year-old man consulting for a progressive fall of visual acuity with hemeralopia. Eye fundoscopy showed regions of confluent rounded chorioretinal atrophy. The visual field and retinal angiography were altered. A high level of plasma ornithine (629 nmol/mL) was detected and a diagnosis of gyrate atrophy of the retina and choroid was made. The patient was treated with high dose Pyridoxine supplement (300 mg/d for 6 months) and the ornithine level of his serum was successfully reduced.

**Conclusion:**

The exact mechanism of chorioretinal atrophy in hyper-ornithinemia is not known and a small percentage of the affected people respond to Vitamin B6 supplementation.

## Background

Gyrate atrophy (GA) of the Choroid and Retina was first described by Fuchs in 1896.[1] Human hereditary deficiency of ornithine aminotransferase (OAT) activity is transmitted as an autosomal recessive trait,[2] and results in 10 to 20-fold increased level of plasma ornithine and is shown to be associated with GA.[3] The initial complaint of decreasing visual acuity and night vision is followed by the appearance of sharply demarcated, circular areas of chorioretinal atrophy with hyperpigmented margins in the midperiphery of the fundus. This appears through the first three decades of life and leads to blindness in the fourth to seventh decades. Myopia, posterior subcapsular cataracts, and vitreous opacities may also be present.[4]

Ornithine delta aminotransferase (OAT) is a mitochondrial nuclear encoded pyridoxal phosphate enzyme that catalyzes the interconversion of ornithine glutamate and proline. Gyrate atrophy is a genetic disorders with increased frequency in the Finnish population with an incidence of one case per 50,000 individuals in Finland.[4] Valle in a review in 2001 revealed that amongst the over 150 biochemically documented cases of GA, about one third of them were from Finland and only seven of them (less than 5%) had been responsive to therapy with Vitamin B6 dietary supplementation.[5]

We report a rare case of a GA, in which the patient's high level of serum ornithine was responsive to therapy with vitamin B6 dietary supplement and was reduced to near normal level.

## Case report

**Figure 1:**
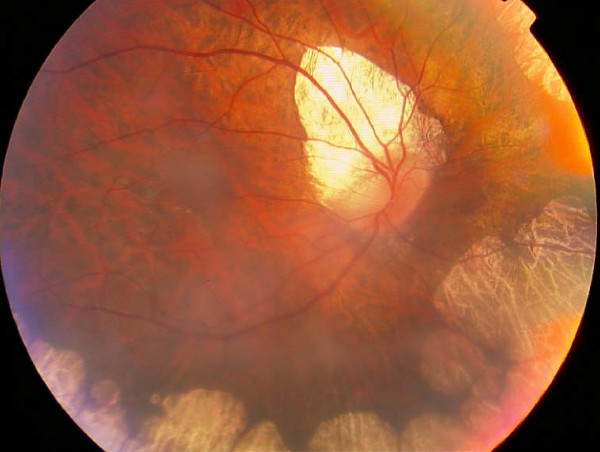
Fundus view of the right eye shows the peripheral and peripapillary chorioretinal atrophic areas. Macula is speared.

A 28-year-old man presented with the complaint of gradual visual loss during the past five years. He had noticed night vision difficulties since the age of 15 years but did not seek medical advice. His visual acuity in each eye was at the level of counting fingers; his refractive error measured -6.00-1.50 × 180 for the right eye and -5.00-2.00 × 170 for the left eye. His best corrected visual acuity was 3/10 in the right eye and 1/10 in the left eye. Bilateral 2+ posterior subcapsular cataracts were present. Fundus examination of both eyes revealed sharply demarcated areas of choroid and retinal atrophy in gyrate shape and involving the midperiphery with the macula not affected. A tilted disc in right eye was also presented (fig 1). Clinical diagnosis identified gyrate atrophy of the choroid and retina. He had mild muscle weakness, but his intelligence and electroencephalography (EEG) were normal. Aminoacid analysis revealed a high serum ornithine level (629 nmol/mL), with the normal range being 28-110 nmol/mL. Arginine, creatinine, lysine and glutamine levels in the serum were within the normal range. He was treated with vitamin B6 at a dose of 300 mg/day for six months. This dietary supplementation successfully reduced his serum ornithine level by more than 50% to 293 nmol/mL.

## Discussion

**Figure 2:**
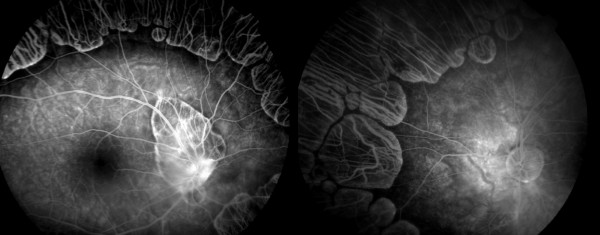
Fluorescein angiogram shows slight leakage from the margins of the choriocapillaris neighboring the atrophic area. The posterior pole was normal.

OTA deficiency is characterized by progressive degeneration of the choroid and retina is inherited an autosomal recessive pattern. In accordance with its ophthalmoscopic appearance, it is called gyrate atrophy of choroid and retina, and there is a mild leakage of fluorescein at the margin of the healthy-appearing retina, where it abuts the gyrate lesions (fig 2). Its main manifestation is a gradual loss of vision, night blindness and tunnel vision, with subsequent development of posterior subcapsular cataract by the second decade,[6] progressive constriction of the visual field and eventual loss of central vision in the fourth to fifth decades of life.[3] The mechanism of GA remains unknown; however, the adverse effects of creatinine or pyrroline-5-carboxilate (P5C) deficiency on retinal function are thought to be a causative factor.[7] The administration of pharmacologic doses of vitamin B6 in a disorder caused by decreased activity of a B6-dependant enzyme is an established procedure.[8] Weleber and Kennaway in a clinical trial of vitamin B6 for gyrate atrophy reported that 3 out of 7 patients responded to oral B6 supplementation with over 50% reduction of serum ornithine.[7] Among approximately 70 Finnish GA cases reported to date, none have been responsive to vitamin B6.[9] Of the remaining studies reported worldwide (from US, Canada, Japan, Italy, Germany, The Netherlands and Israel) only 7 have been reported to have responded to vitamin B6 treatment.[10] Our patient is a rare case of GA in that his serum ornithine level decreased by more than 50% from 629 nmol/mL to 293 nmol/mL after administration of 300 mg/day vitamin B6 as a dietary supplementation.

## Competing interests

The author(s) declare that they have no competing interests.

## Authors' contributions

The authors were involved in patient management or writing of the manuscript.
